# Risk Prediction Score for Chronic Kidney Disease in Healthy Adults and Adults With Type 2 Diabetes: Systematic Review

**DOI:** 10.5888/pcd20.220380

**Published:** 2023-04-20

**Authors:** Alejandra González-Rocha, Victor A. Colli, Edgar Denova-Gutiérrez

**Affiliations:** 1 Centro de Investigación en Nutrición y Salud, Instituto Nacional de Salud Pública, Cuernavaca, México; 2 Facultad de Medicina, Universidad Juárez Autónoma de Tabasco, Tabasco, México

## Abstract

**Introduction:**

Chronic kidney disease (CKD) is an important public health problem. In 2017, the global prevalence was estimated at 9.1%. Appropriate tools to predict the risk of developing CKD are necessary to prevent its progression. Type 2 diabetes is a leading cause of CKD; screening the population living with the disease is a cost-effective solution to prevent CKD. The aim of our study was to identify the existing prediction scores and their diagnostic accuracy for detecting CKD in apparently healthy populations and populations with type 2 diabetes.

**Methods:**

We conducted an electronic search in databases, including Medline/PubMed, Embase, Health Evidence, and others. For the inclusion criteria we considered studies with a risk predictive score in healthy populations and populations with type 2 diabetes. We extracted information about the models, variables, and diagnostic accuracy, such as area under the receiver operating characteristic curve (AUC), C statistic, or sensitivity and specificity.

**Results:**

We screened 2,359 records and included 13 studies for healthy population, 7 studies for patients with type 2 diabetes, and 1 for both populations. We identified 12 models for patients with type 2 diabetes; the range of C statistic was from 0.56 to 0.81, and the range of AUC was from 0.71 to 0.83. For healthy populations, we identified 36 models with the range of C statistics from 0.65 to 0.91, and the range of AUC from 0.63 to 0.91.

**Conclusion:**

This review identified models with good discriminatory performance and methodologic quality, but they need more validation in populations other than those studied. This review did not identify risk models with variables comparable between them to enable conducting a meta-analysis.

**Summary**

**What is already known on this topic?**

Previous reviews found 30 models to identify chronic kidney disease (CKD) in healthy populations, some with good discriminatory performance.

**What is added by this report?**

Our study identified 36 models for risk of CKD in healthy populations and 12 in populations with type 2 diabetes. We found 13 models with good discriminatory performance for healthy populations and 4 for populations with type 2 diabetes.

**What are the implications for public health practice?**

These models could be tools for preventing CKD. Some of them could be the base to develop a tool for use in primary care settings.

## Introduction

Chronic kidney disease (CKD) has been defined as abnormalities of kidney structure or function present for more than 3 months (1). CKD is a public health problem (2–4). According to data from the Global Burden of Disease (GBD) study, in 2017 (4) the prevalence of CKD was estimated at 9.1% globally. Of total mortality, 4.6% of deaths were attributable to CKD and cardiovascular disease (CVD), which was attributable to impaired kidney function.

Type 2 diabetes became the second leading cause of CKD and CKD-related deaths in 2019 (3). Impaired fasting plasma glucose, high blood pressure, high body mass index, a diet high in sodium, and lead were risk factors for CKD quantified in GBD. Approximately 31% of CKD disability-adjusted life years were attributable to diabetes (4).

After automatic reporting of the glomerular filtration rate (eGFR) began, referrals to nephrology specialists by primary care services increased. However, the proportion of appropriate referrals did not change, indicating a need to develop appropriate screenings for CKD (5). Persons living with hypertension, diabetes, or cardiovascular diseases should be screened for CKD; identifying and treating CKD would reduce the burden of kidney disease (6). CKD can be detected early through inexpensive interventions (4).

Echouffo-Tcheugui and Kengne presented a systematic review with 30 models predicting the occurrence of CKD and concluded that some models had acceptable discriminatory performance (7). CKD screening in groups at high risk is likely to be cost-effective. Predictive models that incorporate clinical information systems would facilitate improved treatment allocations and health care management (6,8).

The aim of our study was to identify the existing prediction risk scores and their diagnostic accuracy for detecting CKD in apparently healthy adults and adults living with type 2 diabetes.

## Methods

We followed the methodology proposed by the Cochrane handbook for systematic reviews of Diagnostic Test Accuracy (DTA). The protocol was published at PROSPERO (https://www.crd.york.ac.uk/prospero/), registration number CRD42021252888.

A search strategy was designed for the following databases: Cochrane Library, Medline/PubMed, Embase, Latin American and Caribbean Health Sciences Literature (LILACS), Cumulative Index to Nursing and Allied Health Literature (CINAHL), PsycInfo, Trip Database, Epistemonikos, and Health Evidence. We used the medical subject heading (MeSH) term “renal insufficiency, chronic” and the terms “risk models” and “predictive models,” which were validated in a pilot search. The detailed search strategy is in the Appendix. The databases used mostly had artificial intelligence that helped to mix these terms with similar terms. All the records were screened by title and abstract, then assessed by full text. We finally selected the ones that met all the selection criteria. The screening process included the reference list of the studies included in this review, other similar reviews, and a manual search of other studies identified for the authors in previous searches.

### Study selection

The inclusion criteria included cohort and cross-sectional studies without language restrictions. The search was intentionally limited from May 2011 to November 2021 to update the information provided in previous reviews.

Studies that included healthy adults and adults living with type 2 diabetes were incorporated. The exclusion criteria were studies where the database used was from hospitalized patients with an initial diagnosis of CKD, and patients living with type 1 diabetes.

Included articles had to report models as a risk assessment tool that predicted CKD in healthy adults or adults living with type 2 diabetes. In this review we excluded predictive models of mortality, progression of CKD, and machine learning technology.

Major outcomes that we sought were area under the receiver operating characteristic curve (AUC) or C statistic to predict the presence or occurrence of CKD in healthy adults and adults with type 2 diabetes. Secondary outcomes that we looked for were sensitivity and specificity to predict the presence or occurrence of CKD in healthy adults and adults with type 2 diabetes. Studies that we included compare their models with reference standards eGFR, albuminuria, or proteinuria.

**Figure 1:**
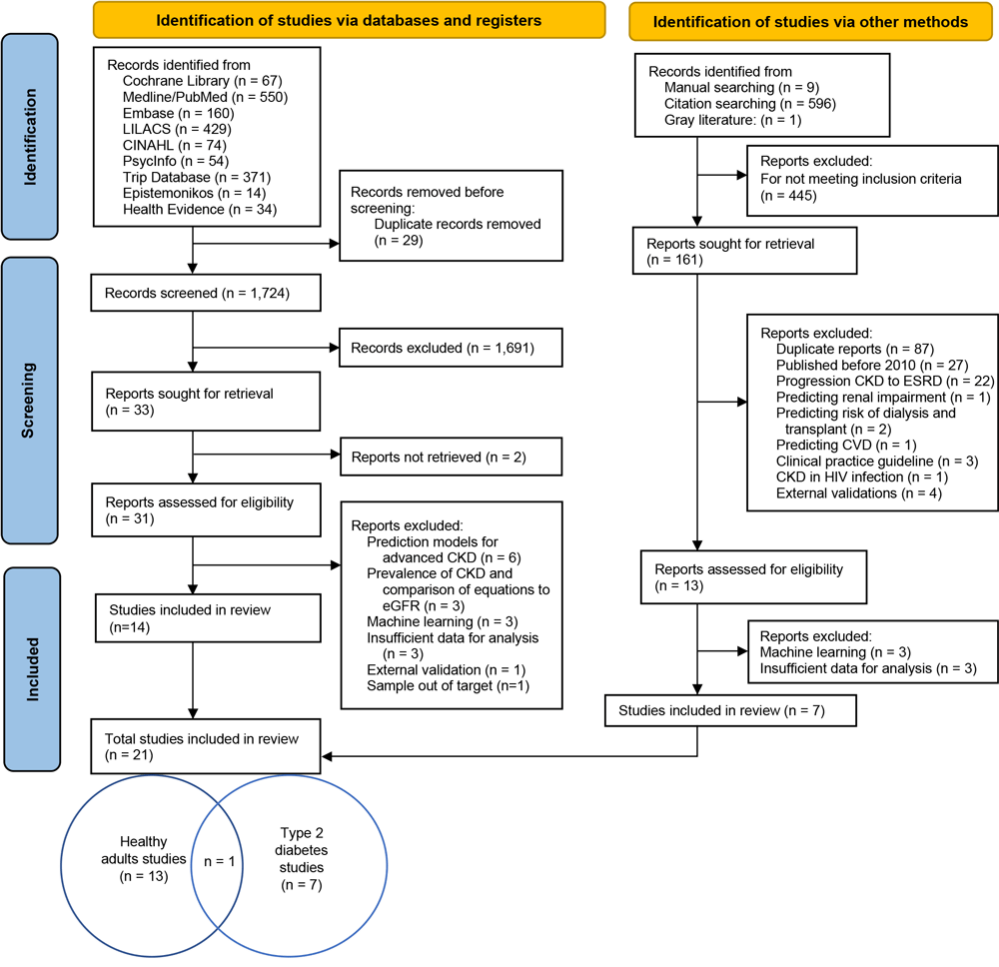
Selection of studies process for analysis of chronic kidney disease (CKD) in healthy adults and adults living with type 2 diabetes. Abbreviations: CINAHL, Cumulative Index to Nursing and Allied Health Literature; CVD, cardiovascular disease; eGFR, glomerular filtration rate; ESRD, end-stage renal disease; LILACS, Latin American and Caribbean Health Sciences Literature.

Two authors of this review (A.G.-R. and V.C.) independently screened titles and abstracts to identify relevant articles. In the first step of this process, reviews were removed, then full texts of the remaining articles were systematically examined for inclusion or exclusion. In the event of disagreement, the participation of the third author (E.D.-G.) was necessary to decide whether to include the article. The selection stages are shown in the flowchart based on Preferred Reporting Items for Systematic Reviews and Meta-Analyses (PRISMA) guidelines (Figure 1).

The information extracted was the design of the studies, type of population studied, type of prediction model and its variables, type of statistical analysis, type of reference standard, and outcomes. We also separated studies by training, development, and external validation models. The data were obtained in duplicate by V.C. and A.G.-R. and corroborated by E.D.-G. The extraction and analysis were performed by separating healthy populations and type 2 diabetes populations; the results are presented as separate groups.

Two reviewers (V.C. and A.G.-R.) independently assessed the risk of bias with Quality Assessment of Diagnostic Accuracy Studies 2 (QUADAS-2), guided by the Cochrane Handbook for Systematic Reviews of Diagnostic Test Accuracy. We used the software tool RevMan 5.4 (Cochrane).

The QUADAS-2 tool evaluated 4 principal domains: 1) patient selection, 2) index test, 3) reference standard, and 4) flow and timing. The applicability concerns were evaluated in 3 domains: 1) patient selection, 2) index test, and 3) reference standard. Each potential bias and concern was graded as high, low, or unclear risk. Risk of bias was evaluated by A.G.-R. and V.C.

### Statistical analysis and data synthesis

To synthesize the information, we divided it by population — healthy population and type 2 diabetes population — and looked for homogeneity in the baseline characteristics of the participants of the studies and the possible risk factors. Nevertheless, because of the heterogeneity of metrics and variables used to assess the predictive ability of CKD risk models, we conducted a qualitative synthesis of the full evidence instead of a meta-analysis.

## Results

### Search results

An electronic search was conducted in May 2021 and updated in January 2022. We identified 2,359 records by searching databases, registers, and other sources; 9 of the studies were identified by doing manual searching, and 1 was from gray literature. From the 2,359 we removed 29 duplicate records. In the screening stage, 1,691 records that did not meet the inclusion criteria were eliminated. Two studies were not retrieved because they were not fully published, leaving 31 studies retrieved for full-text analysis. Of these, 17 were eliminated because they 1) were prediction models for advanced CKD, 2) were about prevalence of CKD, 3) had insufficient data for analysis, or 4) had a sample that mixed type 2 diabetes and type 1 diabetes populations (9). From other methods (reference scanning, manual searching, and gray literature), 7 studies were retrieved. Finally, for the qualitative analysis 21 studies were included: 13 studies (10–22) with prediction models to assess the presence or occurrence of CKD in healthy adults, 7 studies (23–28, and one unpublished paper [A. Raña-Custodio, M. Lajous, E. Denova-Gutiérrez, M. Chávez-Cárdenas, R. Lopez-Ridaura, and G. Danaei, personal communication, 2023]) with prediction model to assess the presence or occurrence of CKD in people with type 2 diabetes, and 1 study including a model for both populations (29). In those studies, we identified 48 different models.

The main characteristics of the risk predictive models for CKD developed in each study are described in Table 1. Fourteen studies were developed by using prospective cohort data, 4 were developed by using cross-sectional data, and 3 were developed by using retrospective cohort data. Of the total studies included, 13 studies’ outcome results were calculated with C statistic, 10 studies with AUC, and 7 studies reported sensitivity and specificity analysis.

**Table 1:** Characteristics of Studies Included in Analysis of Studies of Chronic Kidney Disease (CKD) in Healthy Adults and Adults Living With Type 2 Diabetes

Author and year	Population, total n; outcomes, n (age)	Study design	Type of statistical analysis	Model identification	Variables included	Accuracy predictor
**Healthy adults**						
Al-Shamsi et al (10), 2019	622; 71 (52.4 y)	Retrospective cohort	Fine and gray regression	Full model	Age, sex, diabetes mellitus, hypertension, dyslipidemia, smoking, cardiovascular disease, systolic blood pressure, diastolic blood pressure, total cholesterol, triglycerides, HbA_1c_, eGFR	AUC
				Stepwise model	eGFR, diabetes, cholesterol, HbA_1c_	
Chien et al (11), 2010	5,168; 190 (51.2 y)	Prospective cohort	Cox proportional hazards regression	Clinical model	Age, BMI, diastolic blood pressure (mm Hg), history of type 2 diabetes, history of stroke	C statistic, sensitivity, specificity
				Biochemical model	Age, diastolic blood pressure, history of stroke, uric acid, postprandial glucose, HbA_1c_, urine protein ≥100 mg/dL	
Hao et al (12), 2017	1,310,363; 7,448 (NR)	Retrospective cohort	Multivariable logistic regression	Model derivation^a^	146 clinical variables including demographics, diagnosis of type 2 diabetes, medications, laboratory test results, and resource utilization	C statistic, sensitivity, specificity
	1,430,772; 8,299 (NR)			Model validation		
Hippisley-Cox and Coupland (13), 2010	1,591,884; 23,786 (35–74 y)	Prospective cohort	Cox proportional hazards regression	Final model	Age, ethnicity, deprivation, smoking, BMI, systolic blood pressure, type 2 diabetes, rheumatoid arthritis, cardiovascular disease, treated hypertension, congestive cardiac failure; peripheral vascular disease, NSAID use, family history of kidney disease, systemic lupus erythematosus (in women), and kidney stones (in women)	AUC
Halbesma et al (14), 2011	6,809; 272 (28–75 y)	Prospective cohort	Multivariable logistic regression	Final model	Age, urinary albumin excretion, systolic blood pressure, C-reactive protein, known hypertension	AUC
Kwon et al (15), 2012	2,921; NR (≥19 y)	Cross-sectional	Multivariable logistic regression	NR	Age, sex, anemia, hypertension, diabetes, cardiovascular disease, and proteinuria	AUC, sensitivity, specificity
	External validation 8,166; NR (≥30 y)				Age, sex, anemia, hypertension, diabetes, cardiovascular disease, and proteinuria	
Lee et al (16), 2019	9,080; 734 (51.8 y)	Prospective cohort	Cox proportional hazards regression	Model 1	Sex, BMI, education level, income, fasting plasma glucose, serum albumin	AUC, C statistic
				Model 2	Sex, BMI, education level, income, fasting plasma glucose, serum albumin, Framingham risk score	
				Model 3	Sex, BMI, education level, income, fasting plasma glucose, serum albumin, eGFR, proteinuria	
				Model 4	Sex, BMI, education level, income, fasting glucose, serum albumin, eGFR, proteinuria, Framingham risk score	
Nelson et al (29), 2019	5,222,711; 974,502 (NR)	Cross-sectional	Multivariable logistic regression	Primary model	Age, sex, race, ethnicity, eGFR, history of cardiovascular disease, ever smoker, hypertension, BMI, albuminuria	C statistic
O’Seaghdha et al (17), 2012	2,490; 229 (45–64 y)	Prospective cohort	Multivariable logistic regression	Model 1: clinical model	Age, type 2 diabetes, hypertension	C statistic
				Model 2: clinical model and baseline eGFR	Age, diabetes mellitus, hypertension, baseline eGFR	
				Model 3: model 2 plus measure of proteinuria	Age, diabetes mellitus, hypertension, baseline eGFR, quantitative albuminuria (urine ACR or dipstick proteinuria)	
Saranburut et al (18), 2017	3,186; 271 (25–54 y)	Prospective cohort	Multivariable logistic regression	Model 1 (clinical)^a^	Age, sex, history of diabetes, systolic blood pressure, waist circumference	AUC
				Model 1a^a^	Substitution of waist circumference with overweight (BMI ≥25)	
				Model 1b^a^	Substitution of hypertension for systolic blood pressur	
				Model 2 (clinical plus limited laboratory tests)^a^	Age, sex, systolic blood pressure, diabetic mellitus, GFR category	
				Model 2a^a^	Substitution of systolic blood pressure with hypertension	
				Model 3 (clinical plus full laboratory tests)^a^	Age, sex, systolic blood pressure, diabetic mellitus, GFR, uric acid, hemoglobin	
				Model 3a^a^	Substitution of hypertension for systolic blood pressure	
				Model 1 (clinical)	Age, sex, history of diabetes, systolic blood pressure, waist circumference or BMI	
				Model 2 (clinical plus limited laboratory tests)	Age, sex, systolic blood pressure, diabetic mellitus, GFR category	
	External validation 1,395 (35–54 y)			Model 1 (clinical)	Age, sex, history of diabetes, systolic blood pressure, waist circumference	
				Model 2 (clinical plus limited laboratory tests)	Age, sex, systolic blood pressure, diabetic mellitus, GFR category	
Thakkinstian et al (19), 2011	3,459; 626 (≥18 y)	Cross-sectional	Multivariable logistic regression	Model 1	Age, diabetes, hypertension, history of kidney stones	C statistic
Umesawa et al (20), 2018	58,855; 7,500 (40–74 y)	Prospective cohort	Multivariable logistic regression	Simple risk prediction	Age, eGFR, proteinuria, hematuria	C statistic
				Full risk prediction	Age, eGFR, proteinuria, hematuria, BMI, systolic blood pressure, medication for hypertension, glucose tolerance, medication for diabetes mellitus, smoking and alcohol intake	
	External validation 76,152; 8,964 (40–74 y)			Simple risk prediction	Age, eGFR, proteinuria, and hematuria	
				Full risk prediction	Age, eGFR, proteinuria, hematuria, BMI, systolic blood pressure, medication for hypertension, glucose tolerance, medication for diabetes mellitus, smoking and alcohol intake	
Wen et al (21), 2020	3,266; 590 (NR)	Prospective cohort	Multivariable logistic regression	Training: simple clinical model^a^	Sex, waist circumference, systolic blood pressure, diabetes mellitus, education	AUC, sensitivity, specificity
				Best fit model^a^	Sex, systolic blood pressure, diabetes mellitus, education, triglyceride, urine ACR, C-reactive protein	
				Validation: simple clinical model	Sex, waist circumference, systolic blood pressure, diabetes mellitus, education	
				Best fit model	Sex, systolic blood pressure, diabetes, education, triglyceride, urine ACR, C-reactive protein	
Yu et al (22), 2021	10,049 total; male: 4,117; 157 (NR)	Prospective cohort	Cox proportional hazards regression	Sex-specific CKD male model	eGFR, HbA_1c_ standard deviation, uric acid, uric acid standard deviation, blood urea nitrogen, albumin, hemoglobin	AUC
	10,049 total; female: 5,932; 270 (NR)			Sex-specific CKD female model	Age, eGFR, triglycerides, HbA_1c_ standard deviation, uric acid, uric acid standard deviation, blood urea nitrogen, albumin, hemoglobin, age at menarche (if ≥17 years)	
**Adults with type 2 diabetes**						
Blech et al (23), 2011	1,274; 556 (62.6 y)	Cross-sectional	Multivariable logistic regression	Score 1	Age, duration of diabetes, diabetes type, sex, ethnicity	C statistic, sensitivity, specificity
				Score 2	5 single-nucleotide polymorphisms in 5 genes (*HSPG2*, *NOS3*, *ADIPOR2*, *AGER*, *CCL5*), age, duration of diabetes, diabetes type, sex, ethnicity	
Dunkler et al (24), 2015	6,766; 1,079 (≥55 y)	Prospective cohort	Multivariable logistic regression	Laboratory model	Urine ACR, eGFR, albuminuria stage (normo- or microalbuminuria), sex, age	C statistic, sensitivity, specificity
	External validation 8,300 (≥55 y)			Clinical model	Delta–urine albumin-creatinine ratio to progression, eGFR, albuminuria stage, sex, age, race (White, Asian, other), diabetes duration (years, log transformed), fasting LDL (mg/dL), glucose (mg/dl), waist circumference (cm), comorbidities major atherosclerotic cardiac events (myocardial infarction, stable or unstable angina, coronary artery bypass grafting, or percutaneous interventions, including angioplasty, stenting, atherectomy), laser therapy for diabetic retinopathy, peripheral artery disease (peripheral arterial angioplasty, limb or foot amputation), stroke or transient ischemic attack, number of antihypertensive drugs prescribed	
Jardine et al (25), 2012	7,377; 2,715 (NR)	Prospective cohort	Cox proportional hazards regression	eGFR ACR model	eGFR, ACR	C statistic
				Final risk prediction model	Ethnicity, eGFR, ACR, systolic blood pressure, hypertension treatment, HbA_1c_ level, diabetic retinopathy, waist circumference	
	External validation 11,140 (NR)				eGFR, urinary ACR, systolic blood pressure, HbA_1c_ level, diabetic retinopathy, blood pressure–lowering treatment at baseline, Asian ethnicity, waist circumference	
Low et al (26), 2017	1,582; 679 (NR)	Prospective cohort	Multivariable logistic regression	Training data set^a^	Log urinary ACR (mg/g), systolic blood pressure (per 10 mm Hg), HbA_1c_, eGFR, (per 5 ml/min/1.73m^2^), LDL cholesterol (mmol/L), age (per 10 years increase)	AUC, sensitivity, specificity
				Test data set	Log urinary ACR, systolic blood pressure, HbA_1c_, eGFR, LDL cholesterol, age (per 10 years increase)	
Nelson et al (29), 2019	5,222,711; 974,502 (NR)	Cross-sectional	Multivariable logistic regression	Diabetic model	Age, sex, race, ethnicity, eGFR, history of cardiovascular disease, ever smoker, hypertension, BMI, albuminuria, diabetes medications (insulin vs only oral medications vs none), HbA_1c_ values, and the interaction between diabetes medications and HbA_1c_ values	C statistic
External validation	2,253,540; 367,159 (NR)			External validation model		
Raña-Custodio et al (unpublished data)^b^	18,148; 1,617 (60.5 y)	Prospective cohort	Multivariable logistic regression	Office equation	Sex, age, BMI, current tobacco smoking and alcohol intake, evolution of diabetes mellitus, current diabetes mellitus treatment scheme (insulin, oral hypoglycemics, or both), prevalent microvascular complication of type 2 diabetes (retinopathy, diabetic foot, neuropathy, or stroke), square of age and female current smoker	C statistic
				Laboratory risk score	Sex, age, BMI, current tobacco smoking and alcohol intake, family history of CKD (defined as any prevalent history of CKD among any first-degree relative), history of hypertension, evolution of diabetes mellitus, current diabetes mellitus treatment scheme (insulin, oral hypoglycemics, or both), microvascular complication of type 2 diabetes (retinopathy, diabetic foot, neuropathy, or stroke), fasting plasma glucose, HbA_1c_, serum creatinine (isotope dilution mass spectrometry), eGFR (Chronic Kidney Disease Epidemiology Collaboration equation), total cholesterol, triglyceride levels	
Wu et al (27), 2017	4,795; 643 (59.3 y)	Prospective cohort	Multivariable logistic regression	Development model	Sex, BMI, systolic blood pressure, duration of diabetes (years)	AUC
Wysham et al (28), 2020	160,031; 9,973 (NR)	Retrospective cohort	Multivariable logistic regression	DKD	Age, sex, geographic region, insurance type, payer type, adapted diabetes complications severity index, a recorded diagnosis of hypertension, a recorded diagnosis of heart failure, anemia, diabetic nephropathy, CKD stage 1 and 2, time interval with diabetes mellitus	C statistic

Abbreviations: ACR, albumin-to-creatinine ratio; AUC, area under the receiver operating characteristic curve; BMI, body mass index; DKD, diabetic kidney disease; eGFR, (estimated) glomerular filtration rate; HbA_1c_, glycated hemoglobin A_1c_; LDL, low-density lipoprotein; NR, not reported; NSAID, nonsteroidal anti-inflammatory drug.

a Development models.

b A. Raña-Custodio; M. Lajous; E. Denova-Gutiérrez, PhD; M. Chávez-Cárdenas; R. Lopez-Ridaura; G. Danaei, personal communication, 2023.

### Healthy population risk scores

We synthesized information from 14 studies (10–22,29) that developed equations to detect the risk of CKD in healthy populations; 36 different predictive models were identified. Three models used 5 risk factors (11,18,21); the number of factors included in the models ranged from 3 (18) and 146 variables (12). Some of the most common risk factors included for the predictive equation were age, sex, type 2 diabetes (glycated hemoglobin A_1c_ [HbA_1c_], fasting plasma glucose, or history of diabetes), kidney function (eGFR, proteinuria, or albuminuria), cardiovascular disease (systolic blood pressure, diastolic blood pressure, or history of hypertension), and obesity (waist circumference or body mass index). For the reference standard, all studies used eGFR, with the cutoff established by Kidney Disease Improving Global Outcomes (KDIGO) guidelines (1). Additionally, 4 studies (15,18,20,29) conducted an external validation.

In 7 studies (11,12,16,17,19,20,29) the outcome was calculated with C statistics; in 8 studies (10,13–16,18,21,22), with AUC; 4 studies (11,12,15,21) reported sensitivity and specificity. The range of C statistics was 0.6 to 0.9; the range of AUC was 0.6 to 0.9 (Table 2). We identified for the healthy population 11 models in 7 (10,13–16,18,22) reports predicting CKD above 0.8 AUC.

**Table 2:** Accuracy Results of Models From Analysis of Studies of Chronic Kidney Disease (CKD) in Healthy Adults and Adults Living With Type 2 Diabetes

Author and year	Model name/stage	AUC (95% CI)	C statistic (95% CI)	Sensitivity	Specificity
**Healthy adults**					
Al-Shamsi et al (10), 2019	Full model	0.90 (0.85–0.95)	NR	NR	NR
	Stepwise model	0.91 (0.85–0.96)	NR	NR	NR
Chien et al (11), 2010	Clinical model	NR	0.76	0.76	0.66
	Biochemical model	NR	0.76	0.88	0.51
Hao et al (12), 2017	Model derivation	NR	0.91	62.61 (95% CI, 61.50−63.71)	97.33 (95% CI, 97.30−97.36)
	Model validation	NR	0.87	50.33 (95% CI, 49.25−51.41)	96.60 (95% CI, 96.57−96.63)
Hippisley-Cox and Coupland (13), 2010	Final model (THIN)	Male: 0.87 (0.87–0.88); female: 0.87 (0.87–0.88)	NR	NR	NR
	(QResearch)	Male: 0.87 (0.87–0.88); female: 0.87 (0.87–0.88)	NR	NR	NR
Halbesma et al (14), 2011	Final model	0.84 (0.82–0.86)	NR	NR	NR
Kwon et al (15), 2012	NR	0.87 (0.84–0.89)	NR	89.4 (95% CI, 84.4–93.2)	70.6 (95% CI, 68.90–72.30)
	External validation	0.78 (0.76–0.80)	NR	NR	NR
Lee et al (16), 2019	Model 1	0.63 (0.61–0.65)	0.65 (0.63–0.67)	NR	NR
	Model 2	0.69 (0.68–0.72)	0.72 (0.70–0.74)	NR	NR
	Model 3	0.79 (0.78–0.81)	0.81 (0.80–0.83)	NR	NR
	Model 4	0.81 (0.80–0.83)	0.83 (0.82–0.85)	NR	NR
Nelson et al (29), 2019	Primary model	NR	0.87 (0.82–0.90)	NR	NR
	External validation	NR	0.84 (0.83–0.87)	NR	NR
O’Seaghdha et al (17), 2012	Model 1: clinical model	NR	0.79	NR	NR
	Model 2: clinical model and baseline eGFR	NR	0.81	NR	NR
	Model 3: model 2 plus measure of proteinuria	NR	0.81	NR	NR
Saranburut et al (18), 2017	Model 1 (clinical)	0.72 (0.69–0.75)	NR	NR	NR
	Model 1a	0.72 (0.69–0.75)	NR	NR	NR
	Model 1b	0.71 (0.68–0.74)	NR	NR	NR
	Model 2 (clinical and limited laboratory tests)	0.79 (0.76–0.82)	NR	NR	NR
	Model 2a	0.78 (0.76–0.81)	NR	NR	NR
	Model 3 (clinical and full laboratory tests)	0.80 (0.77–0.82)	NR	NR	NR
	Model 3a	0.79 (0.76–0.82)	NR	NR	NR
	Model 1 (clinical)	0.71 (0.68–0.74)	NR	NR	NR
	Model 2 (clinical plus limited laboratory tests)	0.75 (0.72–0.78)	NR	NR	NR
	External validation: model 1 (clinical)	0.66 (0.55–0.78)	NR	NR	NR
	Model 2 (clinical and limited laboratory tests)	0.88 (0.80–0.95)	NR	NR	NR
Thakkinstian et al (19), 2011	Model 1	NR	Derivative 0.77	NR	NR
	Model 2	NR	Validated 0.74	NR	NR
Umesawa et al (20), 2018	Simple risk prediction	NR	Male: 0.82; female: 0.82	NR	NR
	Full risk prediction	NR	Male: 0.82; female: 0.82	NR	NR
	External validation: simple risk prediction	NR	Male: 0.82; female: 0.81	NR	NR
	Full risk prediction	NR	Male: 0.83; female: 0.81	NR	NR
Wen et al (21), 2020	Training: simple clinical model	0.71 (0.68–0.74)	NR	NR	NR
	Training: best-fit model	0.72 (0.69–0.75)	NR	NR	NR
	Validation: simple clinical model	0.71 (0.68–0.74)	NR	70.49 (95% CI, 63.30–77.00)	65.14 (61.90–68.30)
	Validation: best-fit model	0.72 (0.69–0.74)	NR	56.83 (95% CI, 49.30–64.10)	76.61 (73.70–79.40)
Yu et al (22), 2021	Sex-specific CKD male model	0.93 (0.90–0.96)	NR	NR	NR
	Sex-specific CKD female model	0.95 (0.93–0.97)	NR	NR	NR
**Adults living with type 2 diabetes**					
Blech et al (23), 2011	Score 1	NR	0.56	64.55	46.54
Dunkler et al (24), 2015	Laboratory model	NR	0.67	NR	NR
	Clinical model	NR	0.69	NR	NR
	External validation	NR	0.69	NR	NR
Jardine et al (25), 2012	eGFR plus albumin-to-creatinine ratio model	NR	0.62 (0.61–0.64)	NR	NR
	Final risk prediction model	NR	0.64 (0.63–0.65)	NR	NR
	External validation	NR	0.64	NR	NR
	External validation	NR	0.62	NR	NR
Low et al (26), 2017	Training data set	0.80 (0.77–0.83)	NR	71.4	72.2
	Test data set	0.83 (0.79–0.87)	NR	75.6	72.3
Nelson et al (29), 2019	Diabetic model	NR	0.80 (0.79–0.83)	NR	NR
	External validation	NR	0.81 (0.80–0.82)	NR	NR
Raña-Custodio et al (unpublished data)^a^	Office equation	NR	0.67	NR	NR
	Laboratory risk score	NR	0.71	NR	NR
Wu et al (27), 2017	Development model	0.71 (0.69–0.73)	NR	NR	NR
Wysham et al (28), 2020	DKD model	NR	0.70	NR	NR

Abbreviations: AUC, area under the receiver operating characteristic curve; CKD, chronic kidney disease; DKD, diabetic kidney disease; NR, not reported.

a A. Raña-Custodio, M. Lajous, E. Denova-Gutiérrez, PhD, M. Chávez-Cárdenas, R. Lopez-Ridaura, G. Danaei, personal communication, 2023.

The prediction score with the highest C statistic was the model derivation (12) that included 146 variables (demographics, clinical, medications, and laboratory test results). The 2 highest AUCs were 1) a model developed by using stepwise analysis (10) that included age, sex, type 2 diabetes (yes/no), hypertension (yes/no), dyslipidemia (yes/no), smoking status (yes/no), cardiovascular disease (yes/no), systolic blood pressure (mm Hg), diastolic blood pressure (mm Hg), total cholesterol (mmol/L), triglycerides (mmol/L), HbA_1c_ (%), and eGFR (mL/min/1.73 m^2^); and 2) the sex-specific model (22) that included, for male participants, eGFR, HbA_1c_ standard deviation (%), uric acid, uric acid standard deviation, blood urea nitrogen, albumin, and hemoglobin; and for female participants, age, eGFR, triglycerides, HbA_1c_ standard deviation, uric acid, uric acid standard deviation, blood urea nitrogen, albumin, hemoglobin, and age at menarche (when age at menarche was ≥17 years).

The sensitivity range was 50.3 to 89.4; the highest sensitivity value was from the Kwon et al model (15) that incorporated age, sex, anemia, hypertension, type 2 diabetes, cardiovascular disease, and proteinuria. The specificity values range was 0.51 to 97.3; the highest specificity was the derivation model (12) that included 146 variables.

### Type 2 diabetes risk scores

We synthesized information from 8 studies (23–29 and 1 unpublished paper [A. Raña-Custodio, M. Lajous, E. Denova-Gutiérrez, M. Chávez-Cárdenas, R. Lopez-Ridaura, and G. Danaei, personal communication, 2023]) that developed risk equations for people with type 2 diabetes, analyzing 11 different models. Most of the predictive models used at least 5 risk factors; the number of factors in the models ranged from 5 (23,24) to 16 variables (Raña-Custodio et al, personal communication, 2023). Some of the most common risk factors included for the predictive equation were age, sex, eGFR, and HbA_1c_.

In 6 studies (23–25,28,29, and unpublished study), outcome accuracy was calculated with C statistics; in 2 studies, with AUC (26,27); and 2 reported sensitivity and specificity (Table 2). The range of C statistics was 0.5 to 0.8; the highest AUC was 0.83. The highest C statistic was from the external validation of the diabetic model (29); this model included age, sex, race, ethnicity, eGFR, history of cardiovascular disease, ever smoker, hypertension, body mass index, albuminuria, diabetes medications (insulin vs only oral medications vs none), and HbA_1c_. The range of sensitivity was 64.5 to 75.6; the highest sensitivity was also the external validation of diabetic model. The specificity range was 46.5 to 72.3; the highest specificity was the model developed test data set (26).

### Methodologic quality of included studies

**Figure 2:**
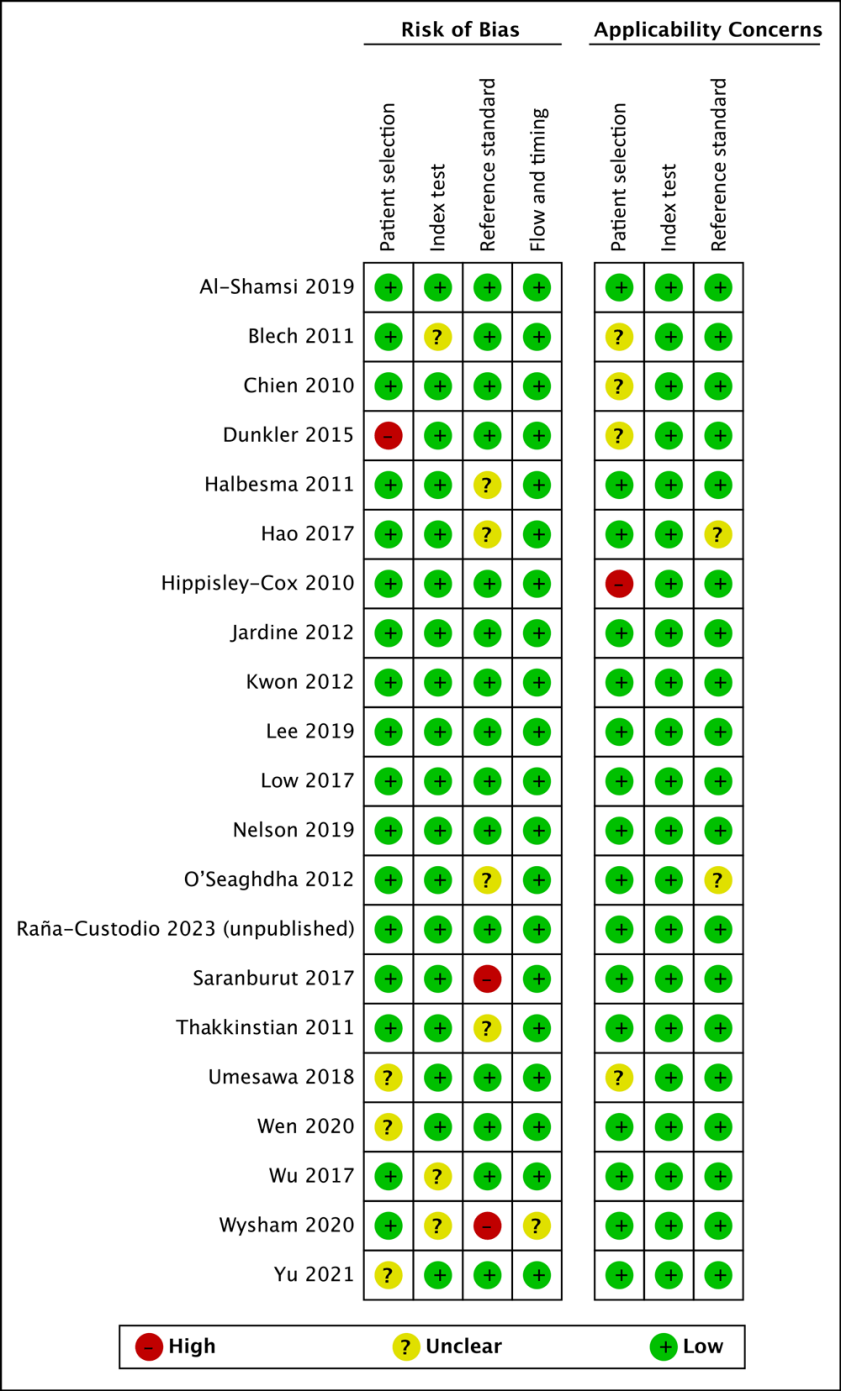
Methodologic quality summary and graph for analysis of studies of chronic kidney disease (CKD) in healthy adults and adults living with type 2 diabetes.

Of all studies included in the synthesis, in the patient selection domain 81% had low risk of bias and 76% had low applicability concern (Figure 2). Dunkler et al had unclear concern of bias because the sample included people receiving pharmacologic therapy (24). Hippisley-Cox and Coupland had high applicability concern because the study population had moderate CKD, recorded by kidney transplants and record of kidney dialysis (13). In the index test domain, 86% had low risk of bias and 100% had low applicability concern. In the reference standard domain, 71% had low risk of bias; Saranburut et al used an outcome from a modification of the KDIGO definition (18). Also in the reference standard domain, 90% had low applicability concern. For the flow and timing domain, 95% had low risk of bias.

**Table:** 

Study	Risk of bias				Applicability concerns		
	Patient selection	Index text	Reference standard	Flow and timing	Patient selection	Index text	Reference standard
Al-Shamsi 2019	Low	Low	Low	Low	Low	Low	Low
Blech 2011	Low	Unclear	Low	Low	Unclear	Low	Low
Chien 2010	Low	Low	Low	Low	Unclear	Low	Low
Dunkler 2015	High	Low	Low	Low	Unclear	Low	Low
Halbesma 2011	Low	Low	Unclear	Low	Low	Low	Low
Hao 2017	Low	Low	Unclear	Low	Low	Low	Unclear
Hippisley-Cox 2010	Low	Low	Low	Low	High	Low	Low
Jardine 2012	Low	Low	Low	Low	Low	Low	Low
Kwon 2012	Low	Low	Low	Low	Low	Low	Low
Lee 2019	Low	Low	Low	Low	Low	Low	Low
Low 2017	Low	Low	Low	Low	Low	Low	Low
Nelson 2019	Low	Low	Low	Low	Low	Low	Low
O’Seaghdha 2012	Low	Low	Unclear	Low	Low	Low	Unclear
Raña-Custodio (unpublished data)	Low	Low	Low	Low	Low	Low	Low
Saranburut 2017	Low	Low	High	Low	Low	Low	Low
Thakkinstian 2011	Low	Low	Unclear	Low	Low	Low	Low
Umesawa 2018	Unclear	Low	Low	Low	Unclear	Low	Low
Wen 2020	Unclear	Low	Low	Low	Low	Low	Low
Wu 2017	Low	Unclear	Low	Low	Low	Low	Low
Wysham 2020	Low	Unclear	High	Unclear	Low	Low	Low
Yu 2021	Unclear	Low	Low	Low	Low	Low	Low

## Discussion

The aim of this systematic review was to identify the existing prediction scores and their diagnostic accuracy for detecting CKD. Thus, we identified 48 different predictive models in 21 total studies of healthy people and people with type 2 diabetes. For healthy populations, we analyzed 14 studies presenting 36 predictive scores for CKD and a wide range (4 to 146) of variables considered by each author. Populations with type 2 diabetes were summarized in 8 studies presenting 15 different models with a range of 4 to 16 variables.

Evaluating the accuracy of these models is a cornerstone to find the best but also reachable way to predict the risk of CKD. In our study, we identified for the healthy population 11 models predicting CKD above 0.8 AUC, considered as good discriminatory performance (30).

This review discords with another review (31). However, by using techniques with a specific tool (QUADAS-2), there are predictive models with good accuracy and quality. For example, Al-Shamsi et al (10) presented a stepwise model for a healthy population with an AUC of 0.9 (95% CI, 0.8–0.9) using variables that are simple and reliable in primary care (eGFR, diabetes, cholesterol, and HbA_1c_), with low risk of bias and low applicability concern. Also, Yu et al (22) presented a sex-specific model with AUC over 0.9 for both sexes, with low risk of bias and low applicability concern.

For the population with type 2 diabetes, Low et al (26), with 0.8 AUC, 75.6 sensitivity, and 72.3 specificity, had the highest accuracy, considered as good discriminatory performance. This risk score includes variables log albumin-to-creatinine ratio, systolic blood pressure, HbA_1c_, eGFR, low-density lipoprotein cholesterol, and age, with low risk of bias and applicability concern. The presence of type 2 diabetes is one of the main risk factors for developing CKD; identifying the population at higher risk is vital for public health. In both populations, the risk models with highest accuracy had HbA_1c_ and eGFR variables in common. Chadban et al recognized that blood glucose plays a significant role in the development of CKD (32), and that is shown in the predictive model that includes a variable related to blood glucose.

These models present a wide heterogeneity between the variables included, similar to findings in other reviews (7,31). Regardless, the heterogeneity found between these predictive equations had common variables: age, hypertension-related variables (systolic blood pressure or diastolic blood pressure), body mass index, and diabetes-related variables (history of diabetes, HbA_1c_, glucose). In agreement with other authors (7), we found that using predictive models with feasible variables in primary care could help professionals from this level of health care alert the population at risk. Also, looking through these variables gave us a chance to look at prevention therapies that control the progression of these variables, as reflected in the progression of CKD. CKD risk predictive models should be applied mainly in populations with risk factors for CKD susceptibility, initiation, or progression (33).

To our knowledge, this is the first systematic review of risk prediction models for CKD that looked at a risk of bias with a validated tool for diagnostic accuracy such as QUADAS-2 to test studies. Conducting a risk of bias analysis is important in a systematic review, because after the accuracy of the studies is identified, the methodologic quality plays an important role for future research and for the populations affected. Also, this review presents results for general populations and for populations with type 2 diabetes that are at higher risk for CKD.

As a limitation, this review did not identify risk models with variables comparable between them to conduct a meta-analysis. Therefore, it is not possible to make recommendations for the use of the models in other populations. We suggest that future work validate in different populations the existing scores and obtain comparable data to make recommendations.

To synthetize the existing models in this report, we gave public health researchers and clinicians a wide view of existing models. From the models they can choose the one that best applies to their population with regard to their accuracy and their methodologic quality.

### Conclusions

We synthesized risk models to detect CKD in healthy and type 2 diabetes patients. Of those, 11 models for healthy populations and 3 for type 2 diabetes patients were identified with good discriminatory performance and methodologic quality. The development of these models, using all those different variables, gives a wide observation of the accuracy and the risk factors. The burden of CKD is increasing in both absolute and relative terms; identifying models that can help to predict the risk of CKD could be the first step to prevent CKD and inform the population. These models are important in primary care settings to help identify people at risk and promptly start prevention or treatment. Some of these models had variables easily obtained at primary care services, improving the accuracy in screening the population at risk and referring patients to a specialist as needed. Finally, these tools need to be externally validated to identify their accuracy in other populations, to provide more information to affected populations regarding public policies about the risk of incident CKD.

## Appendix

**Table:** Search Strategies Used for Analysis of Studies of Chronic Kidney Disease in Healthy Adults and Adults Living With Type 2 Diabetes

Database	Search strategy
Cochrane Library	(chronic kidney insufficiency) OR (Chronic kidney disease) AND (Predictive models) AND adults
Medline/PubMed	(((“risk models”[Title/Abstract] OR “predictive models”[Title/Abstract] OR “Algorithm”[Title/Abstract])) AND ((“chronic kidney disease”[Title/Abstract]) OR (chronic renal insufficiency[MeSH Terms]))) AND (2011:2021[pdat])
Embase	(‘chronic kidney failure’ AND ‘risk assessment’ OR ‘predictive accuracy’) AND ‘normal human’
Latin American and Caribbean Health Sciences Literature (LILACS)	(predictive models) OR (clinical decision rules) AND (chronic renal insufficiency) OR (chronic kidney disease) AND (db:(“LILACS” OR “IBECS” OR “CUMED” OR “BINACIS” OR “BDENF” OR “MULTIMEDIA” OR “PREPRINT-MEDRXIV” OR “BBO” OR “BIGG”))
Cumulative Index to Nursing and Allied Health Literature (CINAHL)	((predictive models) OR (clinical decision rules)) AND (chronic renal insufficiency or chronic kidney disease or chronic kidney insufficiency or chronic renal disease)
PsycInfo	((predictive models) OR (clinical decision rules)) AND (chronic renal insufficiency or chronic kidney disease or chronic kidney insufficiency or chronic renal disease)
Trip Database	(title:adults)(title:(predictive models) OR (clinical decision rules) AND (chronic renal insufficiency) OR (chronic kidney disease))
Epistemonikos	(title:((risk models OR predictive models OR Algorithm)) OR abstract:((risk models OR predictive models OR Algorithm))) AND (title:((chronic kidney disease OR chronic renal insufficiency)) OR abstract:((chronic kidney disease OR chronic renal insufficiency)))
Health Evidence	[(chronic kidney disease) OR (chronic kidney failure) AND (Risk model) OR predictive] AND Limit: Date = Published from 2010 to 2021
